# P2Y Receptors in Synaptic Transmission and Plasticity: Therapeutic Potential in Cognitive Dysfunction

**DOI:** 10.1155/2016/1207393

**Published:** 2016-03-16

**Authors:** Segundo J. Guzman, Zoltan Gerevich

**Affiliations:** ^1^Institute of Science and Technology Austria (IST Austria), Am Campus 1, 3400 Klosterneuburg, Austria; ^2^Institute of Neurophysiology, Charité-Universitätsmedizin Berlin, Charitéplatz 1, 10117 Berlin, Germany

## Abstract

ATP released from neurons and astrocytes during neuronal activity or under pathophysiological circumstances is able to influence information flow in neuronal circuits by activation of ionotropic P2X and metabotropic P2Y receptors and subsequent modulation of cellular excitability, synaptic strength, and plasticity. In the present paper we review cellular and network effects of P2Y receptors in the brain. We show that P2Y receptors inhibit the release of neurotransmitters, modulate voltage- and ligand-gated ion channels, and differentially influence the induction of synaptic plasticity in the prefrontal cortex, hippocampus, and cerebellum. The findings discussed here may explain how P2Y_1_ receptor activation during brain injury, hypoxia, inflammation, schizophrenia, or Alzheimer's disease leads to an impairment of cognitive processes. Hence, it is suggested that the blockade of P2Y_1_ receptors may have therapeutic potential against cognitive disturbances in these states.

## 1. Introduction

Adenosine triphosphate (ATP), the general currency in energy conversions within all living cells, was discovered in 1929 in muscle tissue [1, 2]. In the same year, Drury and Szent-Györgyi described that ATP and its metabolite, adenosine, exhibit potent extracellular activity on the heart and coronary blood vessels [3]. Follow-up studies revealed that extracellular purines are involved in several central and peripheral physiological mechanisms [4] and in the early 1970s Burnstock suggested the existence of purinergic neurotransmission with the release of ATP and its actions on purinergic receptors [5, 6]. In the 1980s it was suggested that ATP receptors, the so called P2 receptors, can be pharmacologically separated into two subtypes: the P2X and the P2Y receptors [7]. While P2X receptors are ligand-gated ion channels permeable for Na^+^, K^+^, and Ca^2+^ [8, 9], P2Y receptors are coupled to G proteins and activate different intracellular cascades [10–12].

Eight different P2Y receptors (P2Y_1_, P2Y_2_, P2Y_4_, P2Y_6_, P2Y_11_, P2Y_12_, P2Y_13_, and P2Y_14_) have been identified exhibiting a different sensitivity to ATP (P2Y_11_), ADP (P2Y_1_, P2Y_12_, and P2Y_13_), UTP/ATP (P2Y_2_ and P2Y_4_), UDP (P2Y_6_), or UDP-glucose (P2Y_14_) [13]. P2Y_1_, P2Y_2_, P2Y_4_, P2Y_6_, and P2Y_11_ receptors are coupled to Gq proteins, the activation of which stimulates phospholipase C and subsequent release of Ca^2+^ from intracellular stores and activation of protein kinase C in response to inositol 1,4,5-trisphosphate and diacylglycerol production, respectively [13, 14]. The P2Y_11_ receptor can also couple to Gs stimulating adenylate cyclase and increasing generation of cAMP [15]. P2Y_12–14_ receptors couple to Gi, effectively inhibiting adenylate cyclase and decreasing cAMP production [13].

P2Y receptors are expressed ubiquitously in the body, including the central nervous system (CNS) [16]. In the CNS, they are localized on neurons, astrocytes, oligodendrocytes, and microglia with physiological roles in neurotransmission, neurogenesis, and glial cell communication [5, 17–20] while they are also involved in a number of peripheral pathophysiological processes, including inflammation, ischemia, and pain [21–27].

ATP can be released from different cell types of the brain such as neurons [28, 29], astrocytes [30], and microglia [31, 32] through exocytotic release mechanism [33], connexin/pannexin hemichannels [34], or P2X7 receptors [35]. After release of ATP, it takes approximately 200 ms before it is hydrolyzed to adenosine in the extracellular space by ectonucleotidases [36, 37]. Although it has been suggested that ATP is involved in fast synaptic transmission in the brain via postsynaptic P2X receptors [38–41], this form of depolarization seems to be insufficient to trigger action potentials in the postsynaptic cells suggesting that the main effect of ATP is neuromodulation similar to other classical neuromodulators such as monoamines and acetylcholine [42]. In these neuromodulational effects of ATP P2Y receptors play an important role. In this review we shall overview the main effects of P2Y receptors on synaptic transmission and plasticity with special emphasis on their network effects and therapeutic potentials in cognitive dysfunction.

## 2. Modulation of Synaptic Transmission

### 2.1. Modulation of Neurotransmitter Release

**Figure 1:**
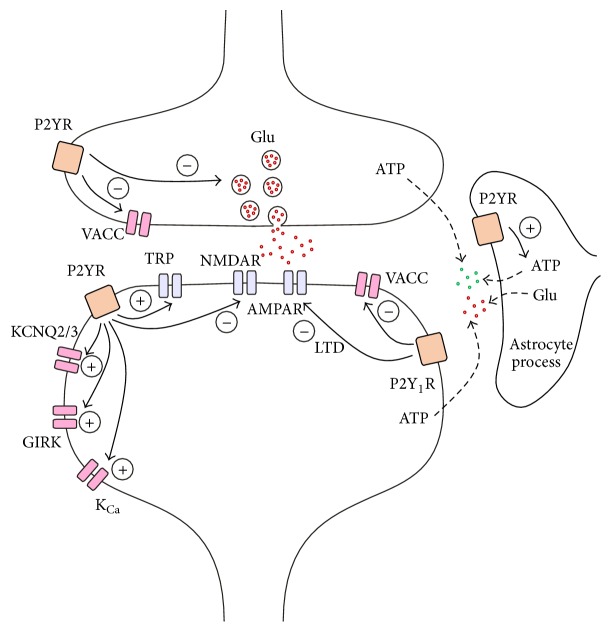
Modulation of excitatory synaptic transmission by P2Y receptors in the CNS. The model synapse shows the main presynaptic and postsynaptic effects of P2Y receptors described in different areas of the brain. For more details see text. AMPAR, AMPA receptor; GIRK, G protein-coupled inwardly rectifying potassium channel; Glu, glutamate; K_Ca_, calcium-activated potassium channel; LTD, long-term depression; NMDAR, NMDA receptor, P2YR, P2Y receptor; P2Y_1_R, P2Y_1_ receptor; TRP, transient receptor potential channel; VACC, voltage-activated calcium channel.

**Figure 2:**
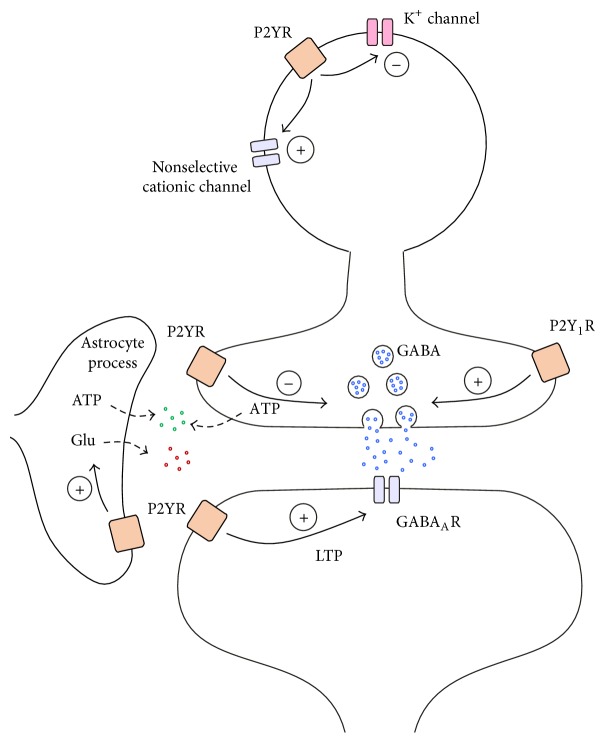
Modulation of inhibitory synaptic transmission by P2Y receptors in the CNS. The cartoon shows an idealized inhibitory (GABAergic) synapse between an inhibitory interneuron and an excitatory principal cell. The effects of P2Y receptors have been described in different brain areas. For more details see text. GABA_A_R, GABA_A_ receptor; Glu, glutamate; LTP, long-term potentiation; P2YR, P2Y receptor; P2Y_1_R, P2Y_1_ receptor.

P2Y receptors have been shown to inhibit the release of a number of neurotransmitters in the CNS [43] (Figures 1 and 2). In the prefrontal cortex, P2Y_1_ receptors have been colocalized with synaptophysin and vGLUT3 suggesting that this receptor subtype is expressed in presynaptic terminals releasing glutamate [44]. P2Y_1_, P2Y_2_, P2Y_4_, P2Y_12_, and P2Y_13_ receptors were shown to inhibit glutamate release from the sensory terminals in the spinal cord [45, 46], from Schaffer collateral synapses of the hippocampus [47–49] and in the cerebral cortex [50]. Underlying this inhibitory effect is most likely a membrane delimited inhibition of N-type voltage-activated calcium channels (VACCs) in the presynaptic terminals via the G*βγ* subunit (see below) [45, 51]. GABA release from basket onto Purkinje cells in the cerebellum was also found to be inhibited by the activation of P2Y_4_ receptors [52]. Noradrenaline release was blocked by P2Y_1_, P2Y_12_, and P2Y_13_ receptors in the spinal cord [46], in the hippocampus [53, 54], and in the cortex [55], possibly via inhibition of VACCs [56]. Similarly, serotonin release in the cortex was decreased after P2Y receptor activation [57]. The modulation of dopamine release by P2Y_1_ receptors seems to be more complex [58–60]; dopaminergic terminals in the prefrontal cortex (PFC) do not contain P2Y_1_ receptors suggesting that multisynaptic mechanisms are involved [44]. In summary, presynaptically located P2Y receptors affect the release machinery of glutamate, GABA, and other neuromodulators. Although the most likely mechanism is the reduction of the release probability due to reduction of presynaptic calcium influx, alternative explanations, such alteration of the fusion machinery or other effects on the pool of vesicles, cannot be entirely discarded.

### 2.2. Modulation of Neurotransmitter Receptors

Activation of P2Y receptors has been shown to modulate numerous membrane receptors and channels in the CNS [61] (Figures 1 and 2). Relatively few data exist demonstrating that P2Y receptors modulate other G protein-coupled receptors. The internalization of the metabotropic glutamate receptor 1 (mGluR1), normally triggered by glutamate, can also be triggered by activation of P2Y_1_ receptors [62].

In addition, various interactions of P2Y receptors with ionotropic receptors are known. Postsynaptically located NMDA receptors were inhibited by P2Y_1_ receptor activation in layer 5 pyramidal cells of the PFC by the G*βγ* subunit [63, 64] (Figure 1). On the contrary, P2Y_4_ receptors exerted positive influence on NMDA receptors. However, this effect required the release of glutamate from astrocytes via P2Y_4_ receptors, which acts on postsynaptic group I mGluRs to enhance the NMDA-mediated current [65]. The sensitivity of postsynaptic GABA_A_ receptors in Purkinje cells was enhanced by P2Y_1_ receptor activation through a Gq-mediated increase in intracellular calcium concentration [66] (Figure 2). P2Y_2_ receptors enhanced currents through Ca^2+^ permeable transient receptor potential vanilloid 1 (TRPV1) channels [67–69]. Although the interaction was found in the peripheral nervous system, TRPV1 are also expressed in the cerebral cortex and hippocampal pyramidal cells [70] and exert a role in synaptic plasticity [71]. P2X receptors have a Ca^2+^ permeability comparable to NMDA receptors [42, 72] that make them potential candidates for the induction of synaptic plasticity. P2X receptors were inhibited by the activation of P2Y_1_ receptors [23, 24].

In summary, P2Y receptors differentially influence the postsynaptic effects of neurotransmitters. Excitatory transmission mediated by postsynaptic NMDA receptors is inhibited by the activation of P2Y receptors, whereas inhibitory transmission through GABA_A_ receptors seems to be enhanced.

### 2.3. Modulation of Voltage-Gated Ion Channels

A typical downstream effect of G protein-coupled receptors is to regulate activity of voltage-gated ion channels. P2Y receptors modulated a number of channels expressed in the CNS and that are critically involved in both synaptic transmission and plasticity [61, 73] (Figure 1). VACCs are a common target of P2Y receptors [74]. Almost all P2Y receptor subtypes have been shown to inhibit N-type VACCs [45, 51, 74–79] by the *βγ* subunit of the G protein binding to the channel in a membrane delimited manner [45, 51]. In addition, P/Q-type VACCs were inhibited by the activation of G*βγ* [74, 80], whereas the inhibition of the L-type channels seems to involve diffusible second messengers and protein kinases activated by G*α* [81–83]. N- and P/Q-type channels are involved in fast presynaptic neurotransmitter release in the CNS; L-type channels are rather localized postsynaptically and regulate dendritic signal integration, neuronal excitability, synaptic plasticity, and gene expression [84]. Thus, P2Y receptors are able to affect all these neuronal processes by interacting with different VACC subtypes (Figure 1).

Different types of potassium channels were shown to be modulated by P2Y receptors [73] (Figure 1). Voltage-gated KCNQ2/3 channels are located in the perisomatic region of pyramidal cell dendrites and are typically closed by activation of Gq-coupled receptors [85]. Almost all subtypes of P2Y receptors have been shown to inhibit KCNQ2/3 channels by activating Gq and intracellular Ca^2+^-dependent mechanisms or by PIP_2_ depletion [86–89]. KCNQ2/3 channels open as neurons approach the threshold for action potential. Because their activation is slow, they are not involved in the repolarization but rather in the afterhyperpolarization thus preventing burst firing of the cell [90]. Their inactivation by a G protein-coupled receptor such as P2Y facilitates membrane excitability and may have a role in the modulation of dendritic integration.

G protein-coupled inwardly rectifying potassium (GIRK1,2,4) channels also contribute to the hyperpolarization of neurons [91] and were found to be activated by P2Y_1_, P2Y_2_, and P2Y_12_ receptors [92–96]. Interestingly, the fast activation of the GIRK channels by G*βγ* and the subsequent hyperpolarization of the membrane were followed in case of P2Y_1_ and P2Y_2_ receptors by a slower inhibition of the channel by G*α* and the subsequent activation of soluble second messengers within the following minutes [92, 95, 96]. While P2Y_4_ and P2Y_6_ receptors only inhibited GIRK channels, P2Y_12_ receptors only opened them [96]. The slower inhibition is also able to reduce the activation of the channel by other GPCRs, for example, by norepinephrine. The fast activation of the potassium channel causes a stabilization of the resting membrane potential around the potassium equilibrium potential, whereas the slow inactivation is able to depolarize neurons expressing this pathway. In addition, neuronal calcium-activated potassium channels were shown to be activated by P2Y_1_ receptors. The increase in intracellular Ca^2+^ upon activation of Gq-coupled P2Y receptors opens these potassium channels and hyperpolarizes the membrane [97–100].

In conclusion, depending on the subcellular expression, P2Y receptors acting on voltage-gated membrane channels are able to inhibit neurotransmitter release, modulate dendritic integration, facilitate neuronal excitability, or affect other various neuronal functions such as synaptic plasticity or gene expression.

## 3. Modulation of Neuronal Circuits

A number of studies investigated the cellular and subcellular distribution of P2Y receptors in the brain. In the hippocampus, P2Y_1_ receptors were located on somata and apical and basal dendrites of pyramidal cells [54, 101, 102]. Additionally, interneurons close to the pyramidal cell layer [101–103] or stratum radiatum interneurons expressing calbindin or calretinin were also stained for P2Y_1_ receptors [104]. While activation of P2Y_1_ receptors did not change the membrane potential in pyramidal cells, their activation on interneurons induced an inward nonselective cationic current likely via activating TRP channels and suppressing the K^+^ conductance (Figure 2) [103, 104]. This depolarized the interneuron membrane by ~10 mV and increased the firing frequency of the cells resulting in increased IPSC frequency in pyramidal neurons [103, 104]. In the cerebral cortex, P2Y_1_ receptors were located on somata and dendrites of pyramidal cells [44, 101], on axon terminals [44], and on parvalbumin containing GABAergic cells in the PFC [44]. They were also described on stellate-like cells in the sensory-motor cortex, medial temporal cortex, and PFC [101]. P2Y receptors seem to have similar roles in the cerebellar cortex, where, together with P2X receptors, they were found to increase the activity of inhibitory basket and stellate neurons projecting onto Purkinje cells and thus decreased the main cerebellar output activity [66, 105, 106]. Therefore, it can be concluded that although purinergic P2Y receptors display an excitatory effect on cell somata on the cellular level, they increase the overall inhibition in two different circuits in the brain by selectively stimulating inhibitory GABAergic interneurons [42].

Although tonic inhibition by volume release of GABA represents one form of cortical inhibition [107], the diversity of GABAergic interneurons in the cortex suggests that their role in neuronal circuits cannot be entirely assigned to a general inhibition. Rather, the role of interneuron firing has to be understood in context of the circuit to which the interneuron type contributes. Interneurons gate the information flow within a circuit and are thus important to coordinate networks [108]. Different interneuron subtypes have been described, involved in different network functions such as dendrite-targeting interneurons (modulation of synaptic efficacy and plasticity of excitatory inputs onto pyramidal cells), interneuron specific interneurons (inhibition of other interneurons), and perisomatic interneurons (synchronization of firing and generation of network oscillations) [109–111]. To understand how P2Y receptors affect neuronal networks, the effects of P2Y receptors on identified interneuron types need to be addressed. Therefore, more information is needed in relation to which interneurons express functional purinergic receptors and how this affects the activity of the network.

The effect of P2Y receptors on neuronal networks was investigated in the hippocampus [112], where P2Y_1_ receptors displayed a stimulatory effect on gamma oscillations in the CA3 area. This was likely mediated by the depolarization of parvalbumin containing perisomatic inhibitory basket cells [103] known to be responsible for the synchronization in the gamma band by rhythmic release of GABA onto pyramidal cells [113]. However, the inhibitory effect of P2X receptors on oscillations seems to be the dominating effect of endogenously released ATP [112]. On the other hand, P2Y receptors play no role in epileptic network activity [114]. Gamma oscillations are involved in higher cognitive functions in the brain by functionally connecting neurons within a local network and between assemblies in different brain areas [115]. In addition, disturbed gamma oscillations have been observed in a line of neuropsychiatric diseases such as schizophrenia, autism spectrum disorders, and Alzheimer's disease (AD) [116, 117]. We suggest that P2Y_1_ receptors, expressed on perisomatic interneurons, are in an ideal position to effectively modulate gamma oscillations and by this mechanism cognitive functions and the development of psychiatric diseases.

In conclusion, P2Y receptors are expressed postsynaptically on dendrites of pyramidal cells and possibly on glutamatergic terminals. In addition, they are present on different types of interneurons in the cortex, including the parvalbumin containing basket cells. It seems likely that, on a network level, P2Y receptors selectively excite interneurons in different cortical areas such as the hippocampus and the cerebellum. Due to the diversity of cortical and hippocampal interneuron subtypes and their physiological functions within the circuit it is of great interest to better understand the cellular distribution of purinergic receptors on different interneuron types.

Gap junctions contribute to network synchronization and are an essential part in the generation and modulation of network activity [118]. Pannexin/connexin hemichannels, on the other hand, are also involved in the release of ATP [119]. Pannexin 1 channels have been shown to be activated by P2Y receptors [120] suggesting that P2Y receptors are able to increase the fast electric communication between cells.

## 4. Involvement in Gliotransmission

The term tripartite synapse describes that, besides the presynaptic nerve terminal and the postsynaptic part of the neuron, processes of astrocytes also participate in the synaptic signaling by bidirectional regulation of neuronal communication [121–123]. Microglia also contact synapses and oligodendroglia have additionally been found to express receptors for neurotransmitters [124, 125]. Astrocytes detect synaptic activity via ionotropic or metabotropic neurotransmitter receptors [126] which cause changes of astrocytic intracellular Ca^2+^ inducing the release of various signaling molecules, such as glutamate, ATP, and D-serine [30]. Gliotransmitters have been shown to act on neurons in a timescale of seconds to minutes to regulate synaptic transmission and plasticity. ATP has a twofold role in the bidirectional neuron-glia communication. First, ATP released from neurons upon activity or during pathological conditions stimulates astrocytes by activation of P2Y_1_ receptors [123] (Figures 1 and 2). Second, ATP released from astrocytes can influence the function of neurons via activation of P2X and P2Y neuronal receptors [127] (Figures 1 and 2). Moreover, P2Y_1_ receptors on neighboring astrocytes are able to amplify the astrocyte stimulation by mediating the propagation of Ca^2+^ waves within the astrocytic network [128]. Bidirectional signaling between glia and neurons occurs by volume transmission [129, 130], and the concentration of the released transmitter drops rapidly from the release site. For that reason, receptors that are involved in neuron-glia-neuron communication, such as purinergic receptors, must have a high affinity for its agonist and a slow desensitization [122].

## 5. P2Y Receptors and Synaptic Plasticity

Several lines of evidence indicate that large amounts of ATP released under pathological conditions such as brain injury or ischemia are able to trigger synaptic plasticity by activation of P2X receptors [131–136]. This plasticity was found to be bidirectional depending on the amount and dynamics of Ca^2+^ influx through P2X channels [131, 132, 137]. Interestingly, ATP released under more physiological conditions is also able to modulate synaptic plasticity acting on P2X receptors. This modulation was shown to be an inhibition of long-term potentiation (LTP) via Ca^2+^-dependent inactivation of NMDA receptors [138, 139] or a facilitation of LTP in the hippocampus [134, 140]. It has been suggested that a moderate and slow increase of intracellular Ca^2+^ generally induces a depression of synaptic transmission via the activation of protein phosphatases and the subsequent internalization of AMPA receptors in the membrane, whereas stronger and faster Ca^2+^ changes induce LTP by activation of protein kinases [136].

Aside from P2X channels, P2Y receptors were also found to have a modulatory role in synaptic plasticity. In the medial habenula nucleus, a region involved in stress, depression, and nicotine withdrawal [141], LTP of AMPA-receptor mediated currents was observed after a 5-minute application of UTP or UDP [142] via activation of presynaptic P2Y_4_ receptors.

In the cerebellum, P2X receptors have been described on Purkinje cells [143, 144], but ATP was not able to evoke membrane conductances suggesting the absence of functional P2X receptors [105, 106]. On the contrary, P2Y receptor activation was shown to evoke Ca^2+^ transients [66, 145]. Accordingly, activation of P2Y receptors induced LTP of the GABAergic transmission between cerebellar interneurons and Purkinje cells via Ca^2+^-dependent increase of GABA_A_ receptor sensitivity [66] (Figure 2).

In the CA1 area of the hippocampus, ATP released from astrocytes upon stimulation resulted in heterosynaptic long-term depression (LTD) of synapses from untetanized neighboring neurons. This was caused by the activation of presynaptic P2Y receptors and the inhibition of glutamate release [149]. Heterosynaptic LTD increases the spatial sharpness of activity-dependent induced LTP. The findings indicate that ATP release from activated astrocytes and the subsequent activation of P2Y receptors are involved in this form of plasticity.

In layer 5 pyramidal cells of the PFC, activation of P2Y_1_ receptors decreased the proportion of cells that develop LTD [64] whereas blockade of P2Y_1_ receptors increased the fraction of plastic cells. In the same cells, pairing a low-frequency presynaptic stimulation with a postsynaptic depolarization induced LTD of excitatory postsynaptic currents [150] (Figure 1). The induction of LTD was dependent on the intracellular increase of calcium via mGluR1s and VACCs. Activation of P2Y_1_ receptors inhibited the induction of LTD. This blockade was absent in the presence of selective antagonists and in mice lacking P2Y_1_ but not P2Y_2_ receptors confirming the sole involvement of P2Y_1_ receptors. P2Y receptors inhibited Ca^2+^ transients in apical dendrites of pyramidal cells suggesting that this is the mechanism responsible for the inhibition of LTD by P2Y_1_ receptors. In addition, ATP, released under hypoxia, was found to inhibit LTD. This effect was mediated by P2Y_1_ receptors because application of a P2Y_1_ receptor antagonist during hypoxia allowed the induction of LTD [150].

These data suggest that effects of P2Y receptors on synaptic plasticity in the hippocampus and cerebellar cortex are different than those found in the PFC. While the effect of P2Y receptors in the hippocampus and cerebellar cortex was to develop both LTP and heterosynaptic LTD, the activation of P2Y_1_ receptors caused an inhibition of LTD in the PFC.

## 6. Pathophysiological Role of Central P2Y Receptors

Among P2Y receptors, P2Y_1_ receptors were suggested as one of the predominant targets of ATP in mediating danger signals in the brain during, for example, ischemia [22, 146, 151] or trauma [148, 152]. One of the main roles of P2Y_1_ receptors under pathological circumstances is the modulation of astrocytic networks by mediation of Ca^2+^ waves and activation of astrocytes upon mechanical injury [153], ischemia [154], or AD [155]. The Ca^2+^ waves evoked by mechanical trauma depressed the activity of neural circuits after mechanical injury [148]. Blockade or deletion of the P2Y_1_ receptors was shown to reduce the infarct volume [22] and cell death in the hippocampus [148] suggesting the mediatory role of the receptor in these processes. P2Y_1_ receptors were found to colocalize with neurofibrillary tangles and amyloid *β* (A*β*) plaques characteristic to AD [156] and reactive astrocytes near A*β* plaques showed enhanced P2Y_1_ receptor mediated Ca^2+^ signaling [155]. The astrocytic hyperactivity could be blocked by inhibiting the release of ATP or by pharmacological antagonism of P2Y_1_ receptors. This suggests that substances that prevent the effect of ATP on P2Y_1_ receptors could be used as therapeutic tools for the treatment of AD [157, 158].

On the contrary, activation of other P2Y receptors was described to have neuroprotective effects in neuroinflammatory processes such as AD [158]. While activation of the P2Y_2_ receptors stimulated neurite outgrow and nonamyloidogenic processing of amyloid precursor protein [159] as well as uptake of A*β* [160], knockdown of the receptors was shown to increase AD pathology [161]. Similarly, P2Y_4_ receptors present on microglia were also found to play a role in the uptake of A*β* [162] and P2Y_12_ receptors were described to stimulate microglial migration towards neuronal damage [163]. Finally, activation of P2Y_13_ receptors on rat primary cerebellar neurons was shown to protect against oxidative stress-induced neuronal death [164].

Emerging evidence indicates that P2Y_1_ receptors are involved in the development of cognitive deficits after traumatic brain injury or focal cerebral stroke (Table 1). Antagonism of P2Y_1_ receptors improved cognitive deficits after controlled cortical impact brain injury [148]. Short (45 min) middle cerebral artery occlusion (MCAO) caused long-lasting sensory-motor and cognitive deficits in mice and rats [147]. While the neurological deficits recovered within weeks, cognitive deficits persisted for long time representing the main clinical problem after ischemia. In P2Y_1_ knockout mice and after antagonism of P2Y_1_ receptors, the cognitive decline after MCAO completely failed, whereas the transient sensory-motor symptoms were still present [147]. Similarly, permanent MCAO induced neuronal damage, astrogliosis, and microgliosis and decreased working and reference memory performances [146]. P2Y_1_ receptor antagonism attenuated the neuronal damage and the cognitive performance without inhibiting the astrocytic or microglial reactivity upon brain injury [146] suggesting that neuronal mechanisms are predominantly involved in the neuroprotective effects of P2Y_1_ receptor antagonism. Application of a selective P2Y_1_ receptor agonist into the medial PFC impaired cognitive performances in working memory and learning tasks [44]. In the same study, stimulation of P2Y_1_ receptors was found to attenuate prepulse inhibition of the acoustic startle reflex without affecting the startle response amplitude [44]. Deficits of prepulse inhibition indicate the reduced capability to filter out unnecessary information that is observed in schizophrenic patients [147].

All together, we suggest that the procognitive and neuroprotective effects provided by P2Y_1_ receptor antagonists may have two components. First, the reduction of glial cell activation may inhibit the network depressing effect of astrocytic calcium waves. Second, the modulation of neuronal communication might influence synaptic transmission, plasticity, and network activity such as neuronal oscillations. The presented data indicate that P2Y receptors, particularly P2Y_1_ receptors, are emerging targets for the treatment of pathological processes that involve cognitive dysfunction. Antagonists of the P2Y_1_ receptor may protect against cognitive impairments after brain injury and have nootropic effects. In contrast, activation of P2Y_2_, P2Y_4_, P2Y_12_, and P2Y_13_ receptors may have a protective effect and might be beneficial in the treatment of neurodegenerative diseases.

## 7. Conclusion

P2Y receptors are activated by ATP released from astrocytes and neurons upon increased neuronal activity or under pathophysiological conditions. They are able to modulate synaptic transmission and plasticity by interactions with voltage-activated calcium and potassium channels, as well as ionotropic receptors. In the hippocampus and the cerebellar cortex, P2Y receptors activate inhibitory GABAergic interneurons playing a key role in timing and organization of principal cell firing. The modulatory effects of P2Y receptors on membrane channels and receptors are sufficient to influence synaptic transmission and plasticity which may sustainably affect the connectivity between different excitatory and inhibitory cell types and thus the network activity in different brain areas. Therefore, P2Y receptors represent important pharmacological targets to treat cognitive dysfunctions and neuropsychiatric diseases, such as Alzheimer's disease and schizophrenia.

## Figures and Tables

**Table 1:** Pharmacological or genetic P2Y_1_ receptor intervention and cognition in animals.

Cognitive domain	Pathological model	Drug/KO	Effect on P2Y_1_R	Appl.	Species	Behavioural task	Effects	Reference
Aversive memory	pMCAO	MRS 2500	Antagonist	i.c.v.	Mice	Passive avoidance test	n.s.	[146]

Fear-based learning	MCAO	MRS 2500	Antagonist	i.c.v	Mice	Contextual fear conditioning test	Reversal of deficit	[147]
	MCAO	P2Y_1_R KO	Knockout			Contextual fear conditioning test	Reversal of deficit	[147]

Recognition memory	pMCAO	MRS 2500	Antagonist	i.c.v.	Mice	Object recognition test	Reversal of deficit	[146]

Spatial memory	pMCAO	MRS 2500	Antagonist	i.c.v.	Mice	Morris water maze	Reversal of deficit	[146]
	Controlled cortical impact injury	MRS 2179	Antagonist	i.c.v.	Mice	Morris water maze	Reversal of deficit	[148]

Working memory	pMCAO	MRS 2500	Antagonist	i.c.v.	Mice	Y-maze test	Reversal of deficit	[146]
		MRS 2365	Agonist	Bilateral infusion into PFC	Rats	DNMTP task	Impairment	[44]

Reversal learning		MRS 2365	Agonist	Bilateral infusion into PFC	Rats	Reversal learning task	Impairment	[44]

Sensory-motor gating		MRS 2365	Agonist	Bilateral infusion into PFC	Rats	PPI of acoustic startle response	Attenuation	[44]

Appl., application; DNMTP, delayed nonmatching to position; i.c.v., intracerebroventricular; KO, knockout; MCAO, middle cerebral artery occlusion; n.s., nonsignificant; P2Y_1_R, P2Y_1_ receptor; PFC, prefrontal cortex; pMCAO, permanent middle cerebral artery occlusion; PPI, prepulse inhibition.
